# The complete mitochondrial genome of *Taiwanaptera montana* (Hemiptera: Aradidae)

**DOI:** 10.1080/23802359.2024.2410439

**Published:** 2024-10-01

**Authors:** Liangpeng Ji, Zhancheng Jia, Xiaoshuan Bai

**Affiliations:** Institute of Life Science and Technology, Inner Mongolia Normal University, Hohhot, China

**Keywords:** Mitochondrial genome, Mezirinae, phylogenetic analysis

## Abstract

*Taiwanaptera montana* is a apterous flat bug found in Yunnan Province, China. This study is the first to sequence, assemble, and annotate the complete mitochondrial genome of *T. montana*. The mitochondrial genome of *T. montana* has a total length of 15615 bp, which is a typical circular DNA, containing 13 protein-coding genes (PCGs), 22 tRNA genes, 2 rRNA genes and a control region, with A + T content of 69.4%. The phenomenon of gene rearrangement was found when the mitochondrial structure was compared with that of other Aradidae. The phylogenetic tree based on 37 mitochondrial genes showed that *T. montana* was most closely related to *Libiocoris heissi*. Aneurinae, Carventinae and Mezirinae are monophyletic groups. In addition, the results also confirmed that Aradinae and Calisiinae diverged earliest and were relatively primitive groups.

## Introduction

*Taiwanaptera montana* Bai, Heiss & Cai, 2017 belongs to Hemiptera, Aradidae, Carventinae, it is a flat bug that lives under the bark or in crevices of fallen trees and feeds on mycelium. Currently, no mitochondrial genome has been reported for the genus *Taiwanaptera*, only morphological descriptions (Bai et al. 2017). To clarify the phylogenetic relationships of Aradidae, we assembly the first complete mitochondria from *T. montana* and we infer a phylogenetic tree, which will provide information for better understanding of the evolutionary relationships among Aradidae.

## Materials and methods

**Figure 1.**
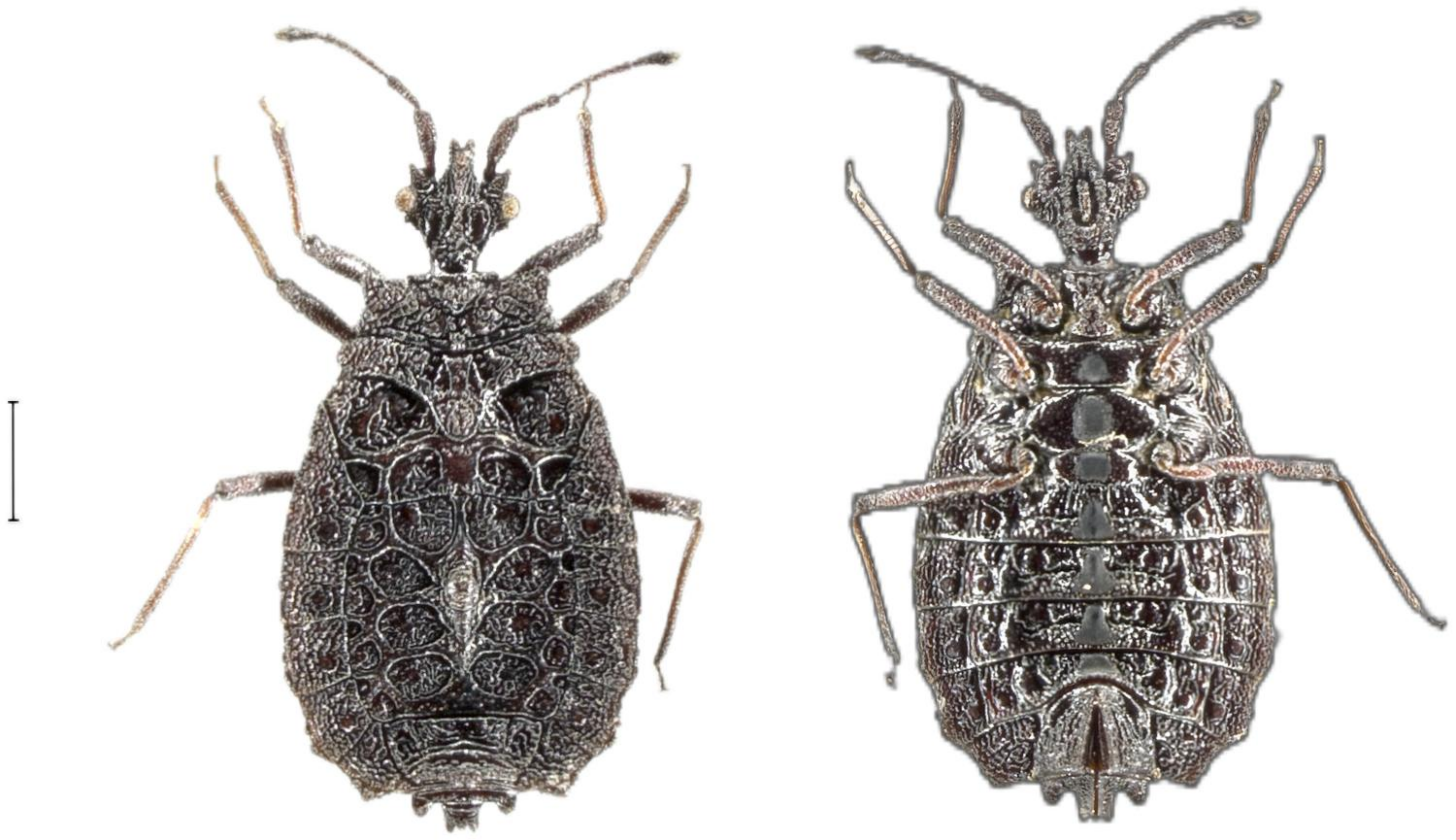
Species reference image of *Taiwanaptera montana*. (the photos of *T. montana* were taken by Liangpeng Ji in the Animal Lab College of Life Science and Technology, Inner Mongolia Normal University, China. The scale is 1 mm).

*T. montana* specimen used in this study was collected in Qushui Township (22.5789°N, 102.2435°E), Pu'er City, Yunnan Province in August 2023. Immediately after collection, the samples were immersed in 95% ethanol and brought back to the laboratory for storage at −18 °C. It was identified as *T. montana* according to the following characteristics: Scutellar ridge distinctly narrower than diameter of lateral sclerites, depressed at middle; antennae about 1.8–1.9 times as long as width of head; postocular lobes granulate; spiracles II–III ventral and not visible from above, IV sublateral and V–VII lateral and visible (Bai et al. 2017) (Figure 1). The voucher number of this specimen is GD-mtb1, and other *T. montana* specimens are deposited in the Insect Collection of Inner Mongolia Normal University (http://bio.imnu.edu.cn/, contact person: Bai XS, baixs2007@aliyun.com).

The identified specimens were photographed and recorded using the KEYENCE VH-S30 B ultra-depth-of-field imager. After rinsing with pure water twice, the head, chest and feet were removed under the anatomical microscope, and then the whole genome DNA was extracted using the TIANamp Genomic DNA Kit according to the instructions. The processed samples were sent to Beijing Berry and Kang Biotechnology Co., Ltd. for sequencing. The second-generation sequencing technology was used, and the sequencing mode was Novaseq 6000-S4-150PE. The returned 4GB Clean data was assembled using SPAdes v3.15.5 (https://github.com/ablab/SPAdes) (Bankevich et al. 2012), and the local blast database was constructed based on the contig sequences obtained using iterative methods during the assembly process. The mitochondrial genome was searched using the BLAST function using the *cox*1 sequence of *Libiocoris Heissi* (NC 030363) as a bait sequence. To check the accuracy of the assembly, reads from clean data were mapped to each sample using Geneious R8 (http://www.geneious.com/) and mitochondrial overlapping regions were obtained, allowing for less than 2% mismatches and a maximum gap of 3 bp, The minimum overlap was 100 bp (Gillett et al. 2014).

The MITOS2 online server (http://mitos.bioinf.uni-leipzig.de/index.py) was used for annotation. The annotation results were confirmed by comparison with homologous sequences in the NCBI database, and then the results were submitted to NCBI. The CGview website (https://cgview.ca/) was used to create a structural map of the mitochondrial genome. MEGA11 (Kumar et al. 2018) was used to calculate the nucleotide composition of genes. Compared with *Drosophila yakuba*, a fruit fly with a mitochondrial genome sequence similar to that of insect ancestors, *trn*Q and *trn*I genes in Aradidae have been rearranged, which may be a unique feature of mitochondrial structure in Aradidae (Liu et al. 2019).

In order to reveal the phylogenetic position of *T. montana*, this study used two species from each of the families Reduviidae, Tingidae, Miridae, and Coreidae as outgroups. Based on 37 mitochondrial genes, the ModelFinder function module (Kalyaanamoorthy et al. 2017) in PhyloSuite v1.2.3 software was used to find the best evolutionary model. Based on the results of ModelFinder (Table S1), We conducted a Bayesian inference method to construct a phylogenetic tree using MrBayes functional module in Phylosuite v1.2.3 (Xiang et al. 2023). The resulting tree was decorated and beautified using the online tool TVBOT (https://www.chiplot.online/tvbot.html) (Xie et al. 2023).

## Results

**Figure 2.**
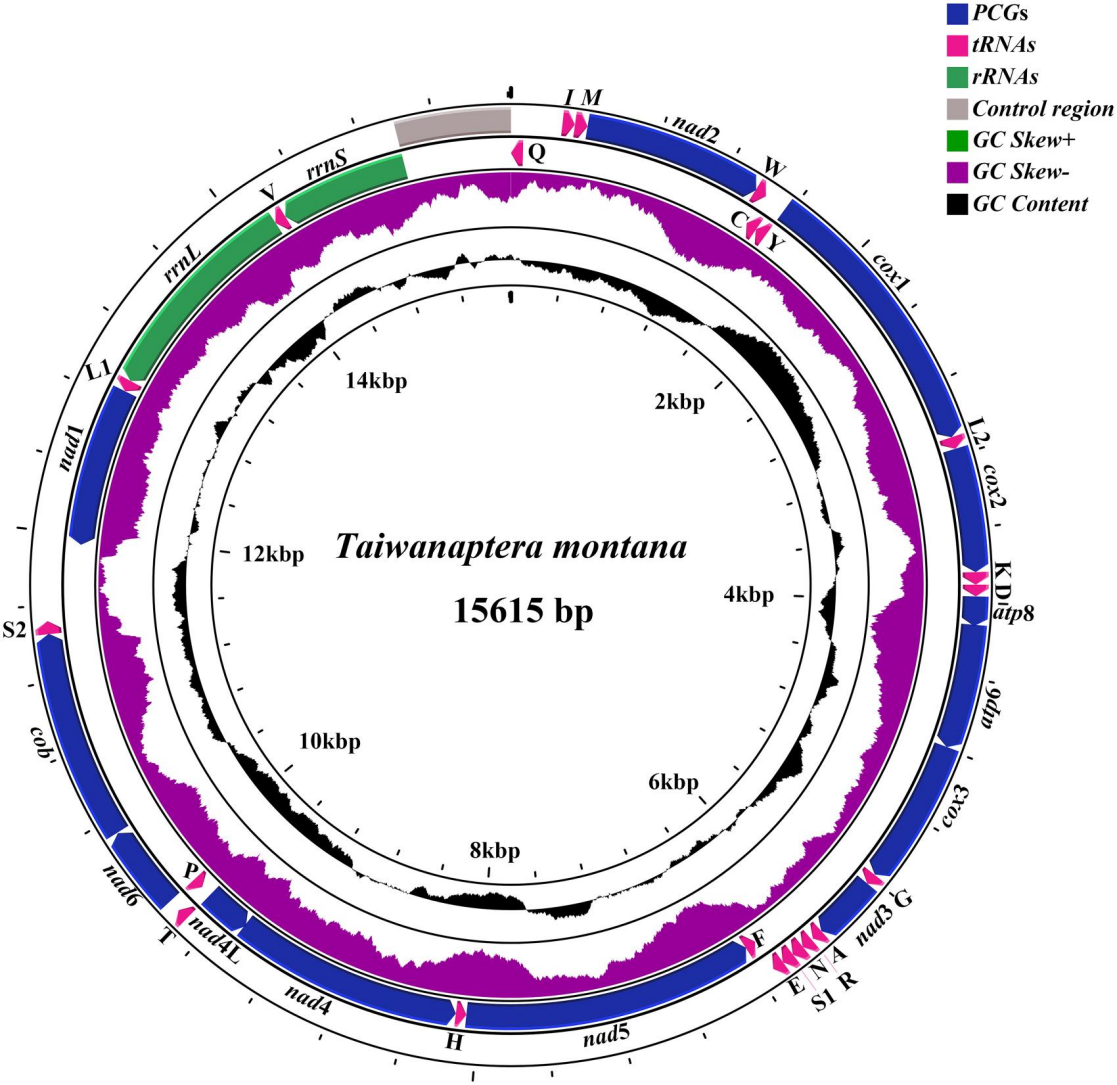
Mitochondrial genome map of *Taiwanaptera montana.*

The mitochondrial genome of *T. montana* was 15615 bp in length, with a base composition of 42.7% A, 26.7% T, 19.1% C, 11.5% G, and A + *T* = 69.4%, showing obvious AT bias (Figure 2). It is a typical closed circular double-stranded structure, which is composed of 37 genes, including 2 rRNA genes, 13 protein-coding genes (PCGs), 22 tRNA genes and 1 noncoding control region (CR). The *T. montana* control region is located between *rrn*S and *trn*Q and is 617 bp in length. Among them, there were 23 genes of J-strand (major strand), including 14 tRNA genes and 9 PCGs. There are 14 genes on the light chain N-strand (minor strand), including 4 PCGs, 8 tRNA genes and 2 rRNA genes. The arrangement and orientation of these genes were consistent with those of most flat bugs, but the *trn*C and *trn*Y genes were shifted compared with those of *Aradus comper* in Aradinae. In contrast to *Aradacanthia heissi* of Calisiinae, inversion of the *trn*C gene and *trn*W gene occurred. Compared with *Drosophila yakuba*, which has a similar mitochondrial genome sequence to the ancestors of insects, the *trn*Q gene and *trn*I gene of Aradidae have been rearranged, which may be a unique feature of the mitochondrial structure of Aradidae.

The 13 PCGs encoded by *T. montana* have a total length of 10917 bp and encode a total of 3639 amino acids. All 11 PCGs used ATN (N = A, T, C, G) as the start codon, and the *nad*2 gene used the rare ATC as the start codon. Both *cox*1 and *nad*1 genes used TTG as the start codon. All nine PCGs have TAA or TAG as stop codons, and *cox*1, *cox*2, *nad*4, and *nad*5 end with T residues.

There are gene gaps or gene overlaps between adjacent genes in the whole coding region of the mitochondrial genome. Except for the control region, there were 10 gene spacers with a total length of 650 bp (gene interval range: 1–425 bp). The longest interval between *nad*1 and *trn*S2 was 425 bp, followed by *trn*Q and *trn*I, and the gene interval was 208 bp. There were 9 gene overlap regions, with a total length of 24 bp (gene overlap range: 1–7 bp). There are two overlaps of 7 bp, located between *atp*8 and *atp*6, and between *nad*4 and *nad*4L.

**Figure 3.**
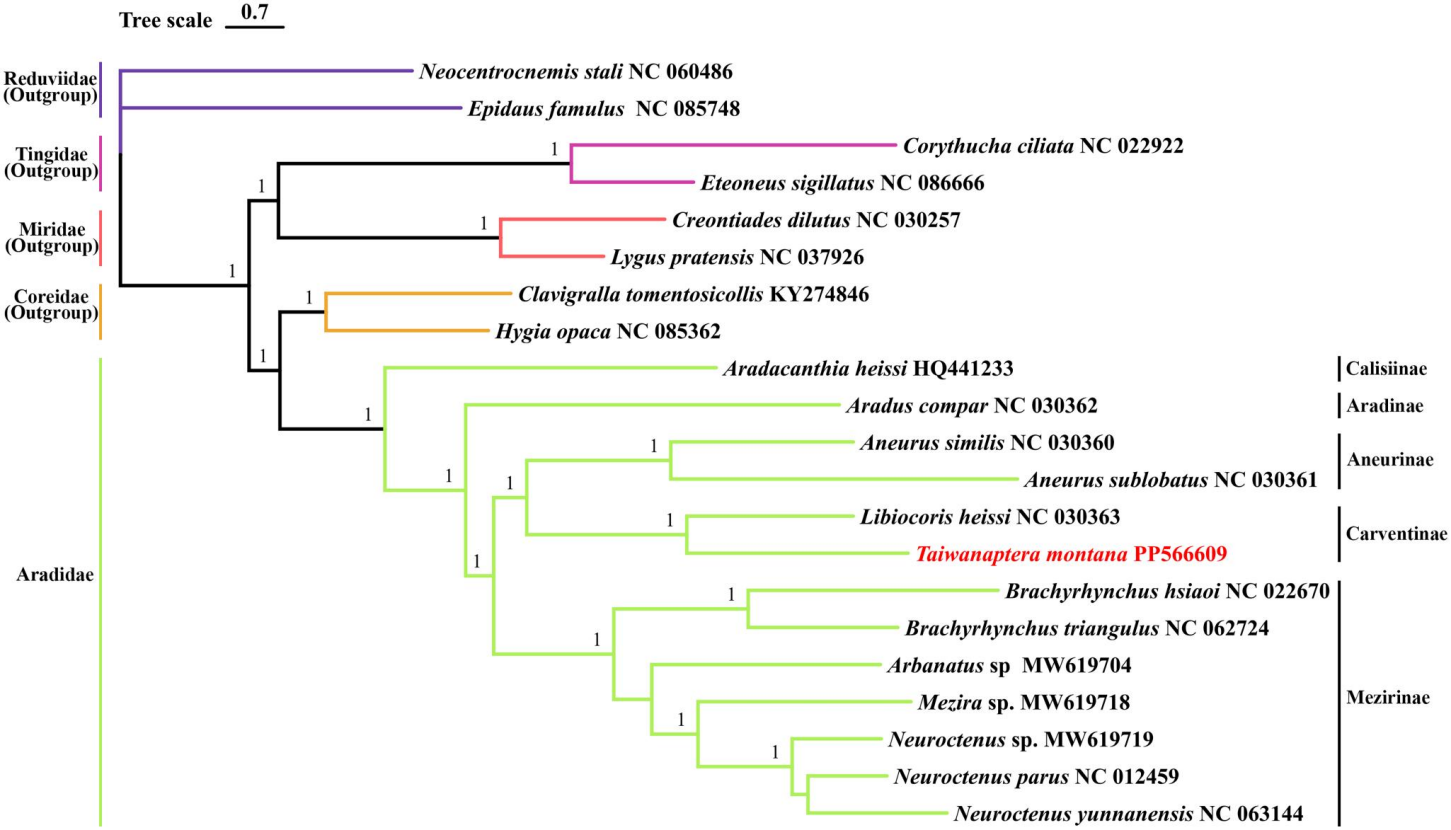
Phylogenetic tree obtained from Bayesian inference (BI) based on complete mitochondrial genomes of 21 species. The optimal zoning and evolutionary models are shown in Table S1. The complete mitochondrial sequences and accession ID were used as follows: *Hygia opaca* NC085362 (Wang et al. 2023); *Neocentrocnemis stali* NC060486; *Lygus pratensis* NC037926 (Tan et al. 2018); *Clavigralla tomentosicollis* KY274846 (Steele et al. 2017); *Epidaus famulus* NC085748; *Corythucha ciliata* NC022922 (Yang et al. 2013); *Eteoneus sigillatus* NC086666; *Creontiades dilutus* NC030257 (Hereward 2016); *Aradacanthia heissi* HQ441233 (Shi et al. 2012); *Aradus compar* NC030362, *Libiocoris heissi* NC030363, *Aneurus similis* NC030360, *Aneurus sublobatus* NC030361 (Song et al. 2016); *Brachyrhynchus hsiaoi* NC022670 (Li et al. 2016); *Brachyrhynchus triangulus* NC062724 (Zhu et al. 2023); *Mezira* sp. MW619718, *Neuroctenus yunnanensis* NC063144, *Arbanatus* sp. MW619704, *Neuroctenus* sp. MW619719 (Ye et al. 2022).

In this study, the four families of the Heteroptera suborder were used as outgroups, and the phylogenetic tree was constructed based on the Bayesian inference (Figure 3). The results supported the monophyly of Aradidae, and showed that *T. montana* and *L. heissi* were closely related, with strong support (BI = 1). Carventinae and Aneurinae were sister groups. It also revealed that *Aradacanthia heissi* and *Aradus compar* were relatively primitive members of Aradidae. In addition, Aradidae is most closely related to Coreidae, and Reduviidae is the more primitive family, which is consistent with the results of previous analysis (Cassis and Schuh 2010).

## Discussion and conclusions

In this article, we report the first complete mitochondrial genome sequence and structural composition data of the genus *Taiwanaptera*, which enriches the bioinformatics database of the mitochondrial Aradidae genome, and analyze the gene rearrangement phenomenon in Aradidae. The results suggest that the mitochondrial genome can be used as a reference for studying the differentiation of taxa (Song et al. 2016). In addition, the phylogenetic analysis based on the complete mitochondrial genome sequence showed that *T. montana* and *L. heissi* were closely related. Aneurinae, Carventinae, and Mezirinae are monophyletic groups. The results were consistent with those of previous studies (Marchal and Guilbert 2016). This provides a good basis for subsequent taxonomic, phylogenetic, and population genetic work on the family Aradidae.

## Supplementary Material

Coverage depth figure.png

## Data Availability

The genome sequence data annotation results of this study are publicly available in GenBank under accession number PP566609. The associated BioProject, SRA and Bio-Sample numbers are PRJNA1092551, SRR28475283 and SAMN40976847, respectively.
