# Supplementary figures and images for: The complete mitochondrial genome of Taiwanaptera montana (Hemiptera: Aradidae)
Source: Mitochondrial DNA B Resour. 2024 Oct 1;9(10):1331–5. doi: 10.1080/23802359.2024.2410439 (PMC11445889; doi:10.1080/23802359.2024.2410439)

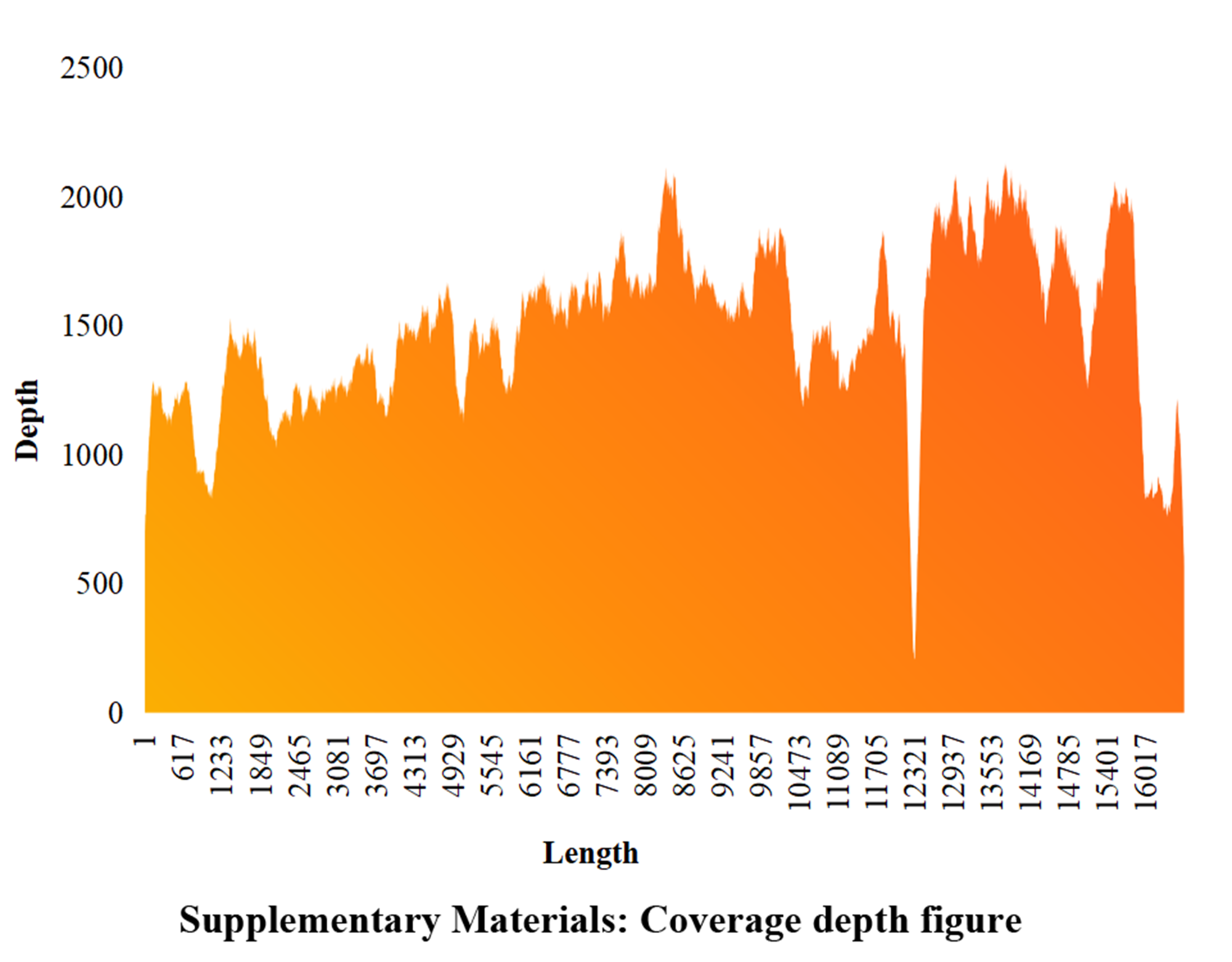

Supplement: Coverage depth figure.png [file TMDN_A_2410439_SM2612.png]
